# Easy labeling of proliferative phase and sporogonic phase of microsporidia *Nosema bombycis* in host cells

**DOI:** 10.1371/journal.pone.0179618

**Published:** 2017-06-22

**Authors:** Jie Chen, Wei Guo, Xiaoqun Dang, Yukang Huang, Fangyan Liu, Xianzhi Meng, Yaoyao An, Mengxian Long, Jialing Bao, Zeyang Zhou, Zhonghuai Xiang, Guoqing Pan

**Affiliations:** 1 State Key Laboratory of Silkworm Genome Biology, Southwest University, Chongqing, P. R. China; 2 Key Laboratory of Sericultural Biology and Genetic Breeding, Ministry of Agriculture, Chongqing, P. R. China; 3 State Key Laboratory of Microbial Technology, School of Life Science, Shandong University, Jinan, P. R. China; 4 College of Life Sciences, Chongqing Normal University, Chongqing, P. R. China; Institute of Plant Physiology and Ecology Shanghai Institutes for Biological Sciences, CHINA

## Abstract

Microsporidia are eukaryotic, unicellular parasites that have been studied for more than 150 years. These organisms are extraordinary in their ability to invade a wide range of hosts including vertebrates and invertebrates, such as human and commercially important animals. A lack of appropriate labeling methods has limited the research of the cell cycle and protein locations in intracellular stages. In this report, an easy fluorescent labeling method has been developed to mark the proliferative and sporogonic phases of microsporidia *Nosema bombycis* in host cells. Based on the presence of chitin, Calcofluor White M2R was used to label the sporogonic phase, while *β-*tubulin antibody coupled with fluorescence secondary antibody were used to label the proliferative phase by immunofluorescence. This method is simple, efficient and can be used on both infected cells and tissue slices, providing a great potential application in microsporidia research.

## Introduction

Microsporidia are obligate intracellular parasites that infect many animal species, including humans and several commercially important organisms, such as bees, silkworms, chickens and aquatic animal [1–4]. Increasing evidence for microsporidiosis prevalence has indicated a connection between pathogen contamination and human health and food chains [5]. *Nosema bombycis*, the first identified Microsporidia, is the pathogen of silkworm *Bombyx mori* which was found by Louis Pasteur [6, 7]. It causes pébrine disease though horizontal transmission and vertical transmission. Vertical transmission of *N*.*bombysis* via eggs could bring big losses to sericulture, thus has been the quarantine pathogen of sericulture in every country that cultivate silkworms. Furthermore, the parasite not only infect the silkworms but also some other insects like Drosophila, locusts, *Pieris rapae*, etc. [8–10]

The life-cycle of microsporidia can be divided into three phases: the infective phase, the proliferative phase and the sporogonic phase [11]. The infective phase contains the mature spores in the environment and, when external factors are suitable, the spores are activated and infect host cells by impalement with polar tube or by phagocytosis [12]. The size of spores are 1–4 μm on average [13] and oval when observed under a light microscope, which are important parameters in diagnosis. The proliferative phase includes sporoplasm and meront in host cells, without chitin and a fixed form, this stage is hard to observe. The sporogonic phase includes sporonts, sporoblasts and spores, which chitin and proteins gradually accumulate along the spore wall [11]. Microsporidia infection is usually detected by microscopy examination against spores, in combination with a series of methods—Giemsa, Gram, and Gram-chromotrope staining, fluorescent stains with Calcofluor White M2R, antibody against spore structure or Fluorescence *in situ* hybridization (FISH) techniques, and stains like Grocott’s methenamine silver, carbol-fuchsin, Heidenhain’s iron hematoxylin, periodic acid–Schiff or Luna stains—were used to make observations [14–20]. However, most of these methods detect mature spores and cannot distinguish different stages of microsporidian cells. Due to the small size and intracellular location of microsporidia, most structures and developmental stages of these parasites have been visualized by Transmission Electron Microscopy (TEM) or Scanning Electron Microscope (SEM), especially as the proliferation stages are difficult to observe by light microscope [11, 21].

Both the cellular activities and protein functions in intracellular stages of microsporidia are significant and interesting. TEM has always be the most useful tool to identify early stage infections and most importantly to gather information on cell structure. However, TEM has high technical requirements and not easily used in every laboratory. FISH is well established and can be combined with other dyes for visualizing microsporidia infection [19, 20, 22]. Recent studies of *Nematocida displodere* infection in *Caenorhabditis elegans* have shown an ideal method to characterize the infection life cycle of microsporidia by using FISH to stain parasite rRNA, 4’6-diamidino-2-phenylindole (DAPI) to stain nuclei, and Direct Yellow 96 (DY96) to stain the chitin of parasite spore walls [23]. Here we demonstrated a novel easy method to label the proliferative and sporogonic phase of microsporidia *N*. *bombycis* in host cells. Based on the presence of chitin, Calcofluor White M2R was used to label the sporogonic phase, while the *β-*Tubulin antibody coupled with a fluorescence marked secondary antibody for labeling the proliferative phase. Then we used this method to display the location of NbSWP12 in the intracellular phase. Our study provides a great application for increasing our understanding of *Nosema* infection at the cellular and tissue level.

## Materials and methods

### Parasite and host

Microsporidia *N*. *bombycis* CQ 1 was isolated from silkworm *Bombyx mori* in Chongqing China and conserved in the China Veterinary Culture Collection Center (CVCC No. 102059). *N*. *bombycis* strains were propagated from silkworms and maintained in the laboratory. Purified spores were obtained using the discontinuous density gradient centrifugation method as previously described [24]. Silkworm *B*. *mori* Dazao strain was maintained in the Gene Resource Library of Domesticated Silkworm (Southwest University, Chongqing, China). Silkworm embryo cells (BmE) were gifted from Professor Pan and cultured in Grace's medium supplemented with 10% fetal bovine serum at 28°C [25].

### Genomic DNA extraction

Purified spores (1X 10^9^) of *N*. *bombycis* were washed three times with sterile water and then suspended in 400 μL of 2% Cetyltrimethyl Ammonium Bromide (CTAB) buffer: 4 g CTAB, 16.36 g NaCl, 20 mL of 1 mol/L Tris-HCl (pH 8.0), 8 mL of 0.5 mol/L EDTA, 400 μL of 0.2% *β*-mercaptoethanol. 0.4 g acid-washed glass beads (Sigma, diameter: 425–600 μm) were added and samples were disrupted in a vortex oscillator at 4°C for 5 min. After resting for 10 minutes, samples were incubated with 20 μL of 20 mg/mL Proteinase K at 55°C for 4 h. Genomic DNA was then extracted with 300 μL of a phenol-chloroform mixture. The supernatant was isolated by centrifugation at 12,000 g for 10 min. Isopropyl alcohol was added to precipitate the genomic DNA at -80°C for 20 min. Precipitates were isolated by centrifugation at 12,000 g for 5 min, followed by washing with 70% ethyl alcohol. After allowing the DNA to air-dry, the genomic DNA was dissolved in sterile water and stored at -20°C.

### Amplification of the *β-tubulin* ORF

*β*-Tubulin is a major component of microtubules. It localizes to the cell membrane in many organisms, but there is no localization data for microsporidia. The *β-tubulin* gene of *N*. *bombycis* (GenBank No. EOB14994.2) was amplified using genomic DNA of *N*. *bombycis* spores by PCR for 30 cycles of 94°C for 15 s, 55°C for 30 s, and 68°C for 1 min using the forward primer 5’-CTGGATCCATGAGAGAAATTATT-3’, containing a Bam HI restriction site (GGATCC), and the reverse primer 5’-CGGTCGACATATACCCTTTAATT-3’ containing a Sal I restriction site (GTCGAC). PCR products were cleaned (Omega) and cloned into pCold I vector (Takara) and transformed into *E*. *coli* DH5α competent cells. The identified pCold I-*β-tubulin* vector was sequenced by Invitrogen (Shanghai, China).

### Protein expression, purification and polyclonal antibody production

pCold I-*β-tubulin* vector was transformed into *E*. *coli* Rosetta and cultured in LB medium at 37°C to OD_600_ = 0.6. The recombinant *β*-Tubulin protein expression was induced by adding 0.2 mM IPTG for 6 h at 37°C. The protein was then purified with a Ni^2+^-nitrilotriacetic acid column (GE Healthcare) based on the manufacturer’s instructions. Mice and rabbit were used to generate antiserum by immunizing with recombinant *β*-Tubulin protein homogenized with Freund’s adjuvant (V/V = 1:1, Sigma-Aldrich). After four weekly immunizations, antiserums were collected and stored at -20°C.

### Immunoblotting

10^9^ mature spores of *N*. *bombycis* suspended in 400 μL of lysis buffer (Beyotime) were mixed with 0.4 g of glass beads (212–300 μm, acid-washed, Sigma-Aldrich) and disrupted by violent oscillation. After centrifugation at 12,000 g for 10 min, the supernatants were isolated and measured for immunoblotting. Proteins were separated on a 12% SDS-PAGE and transferred to PVDF membrane (Roche). The membranes were treated as follows: blocking for 1 h at 37°C in Blocking Buffer (Beyotime), washing three times, incubating with a 1:9,000 dilution of *β*-Tubulin antiserum for 1 h at 37°C, washing three times, incubating with 1:8,000 peroxidase-labelled goat anti-mouse IgG (Roche) for 1 h at 37°C, washing three times, and incubating with Pierce^™^ ECL Western Blotting Substrate for 1 min, followed by imaging using a Azure Biosystems C300 imaging system.

### Preparation of cells for fluorescence microscopy

Infected cells or tissue slice were fixed and permeabilized as previously described [26, 27]. Samples were washed three times with PBS + 0.1% Triton X-100 (PBST) and blocked in PBST containing 10% goat serum and 5% BSA for 1 h at 37°C. After three washes, samples were incubated for 1 h at 37°C with *β-tubulin* antiserum or negative serum (diluted 1:200 in blocking solution) containing 2% Triton X-100. After washing with PBST, the samples were maintained in darkness for 1 h at 37°C with Alexa Fluor^®^ 488 or 594 conjugate Goat anti-Mouse IgG (Thermo Fisher). Samples were then washed in PBST and stained for 5 min in DAPI (4’6-diamidino-2-phenylindole, Sigma) for nucleus labeling. After washing with PBST, ProLong^®^ Gold antifade reagents (Thermo Fisher) were added and cells were imaged using an Olympus FV1200 laser scanning confocal microscope.

### Labeling different phases of microsporidian

Chitin was either presence or absence in different phases of microsporidian, which allows an easy labeling method to be utilized. *β*-Tubulin antibodies were used to label *N*. *bombycis* by an indirect immunofluorescence assay. 0.1 μg/mL Calcofluor White M2R (Sigma), which stained chitin, was used to label the sporogonic phase of *N*. *bombycis* [28]. Negative antiserum were used as negative control. After washing with PBST, ProLong^®^ Gold antifade reagents (Thermo Fisher) were added and cells or tissue slice were imaged by an Olympus FV1200 laser scanning confocal microscope.

### Identifying protein locations

In order to evaluate the method in detecting protein locations, we investigate the protein location of NbSWP12 in the intracellular phase. Infected cells were fixed, permeabilized and blocked as described above. Then the samples were simultaneously incubated with NbSWP12 mouse antibody and *β*-Tubulin rabbit antiserum (diluted 1:200 in blocking solution containing 2% Triton X-100) for 1 h at 37°C. After washes, the samples were incubated for an additional 1 h with a 1:2000 dilution of Alexa Fluor^®^ 488 conjugate Goat anti-Mouse IgG and a 1:2000 dilution of Alexa Fluor^®^ 594 conjugate Goat anti-Rabbit IgG (Thermo Fisher) in a dark moist chamber at 37°C. The chitin present in spores was stained with 0.1 μg/mL Calcofluor White M2R (Sigma) for 5 min at room temperature. Then the samples were washed and observed using a confocal laser scanning microscope.

## Results

### Expression of *β*-Tubulin and immunoblot analysis

**Fig 1:**
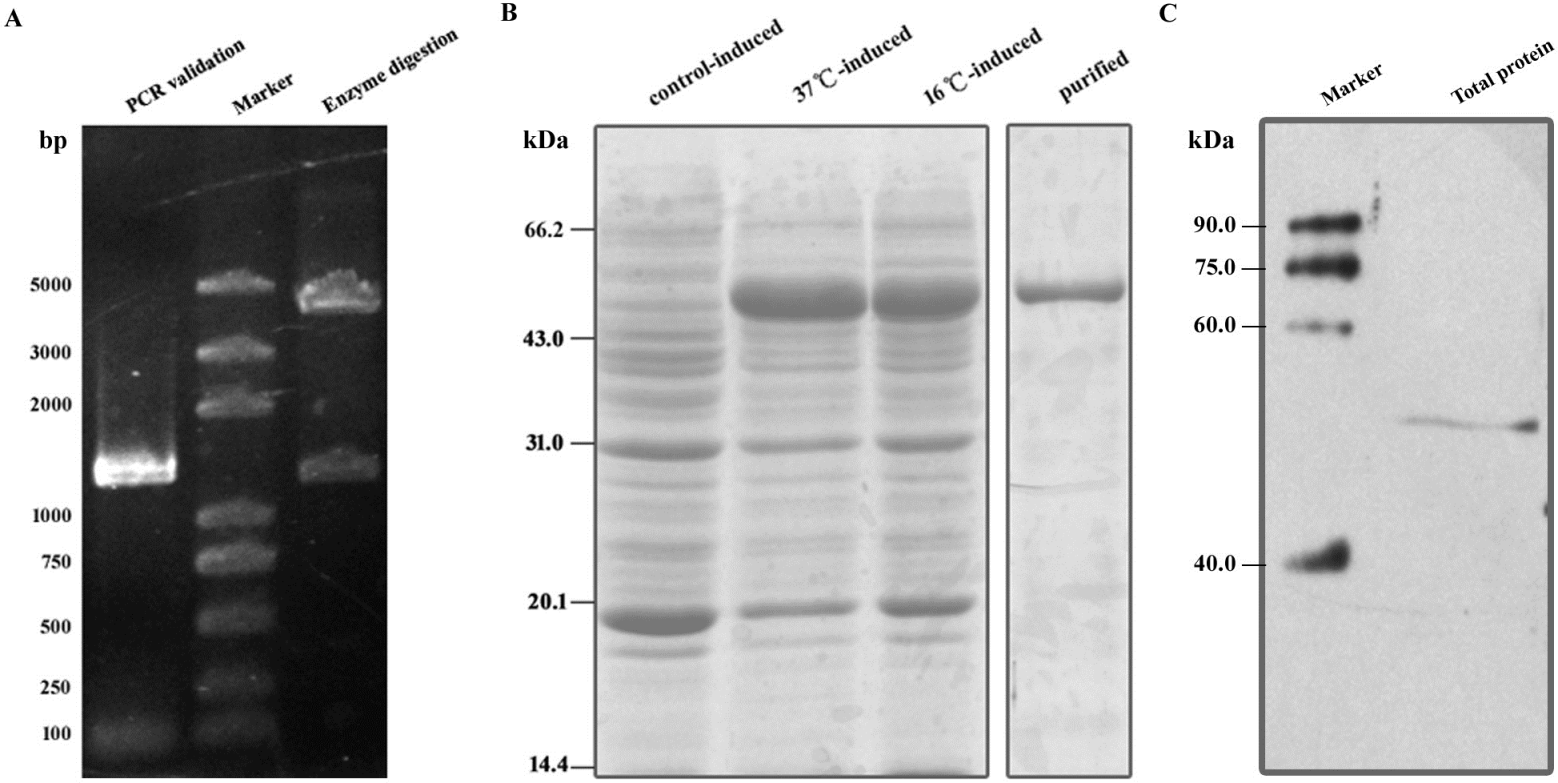
Expression of *β*-Tubulin. (A) Validation of pCold I-*β-tubulin* vector by PCR and Bam HI/Sal I enzyme digestion. ~1300 bp products were amplified by PCR or cleaved from recombinant vector. (B) SDS-PAGE of proteins expressed in *Escherichia coli* Rosetta. Recombinant *β-*Tubulin protein was induced to express at 37°C and 16°C. pCold I vector transformed *E*. *coli* Rosetta were induced for expression at 37°C as a control. (C) Immunoblot for *β-*Tubulin in total protein of *Nosema bombycis* mature spore. The antibody recognized a 50 kDa band which was consistent with prediction.

The open reading frame of *β-tubulin* (S1 Text) was amplified by PCR using specific primers on the genomic DNA of *N*. *bombycis*. The PCR products were successfully cloned into a pCold I vector (Fig 1A). After sequencing, a 1326 base pair fragment that coded for 442 amino acids was obtained. The sequence was consistent with the data from the genomic sequence (http://silkpathdb.swu.edu.cn/).

SDS-PAGE analysis indicated that recombinant *β-*Tubulin protein was expressed at a molecular mass of nearly 53 kDa, which was consistent with the deduced size (Fig 1B). After purification, the protein was used to prepare the antibody. Immunoblot assays indicated that the *β-*Tubulin antiserum recognized a 50 kDa protein in the extract of *N*. *bombycis* mature spores (Fig 1C).

### Location of *β*-Tubulin in the intracellular phase

**Fig 2:**
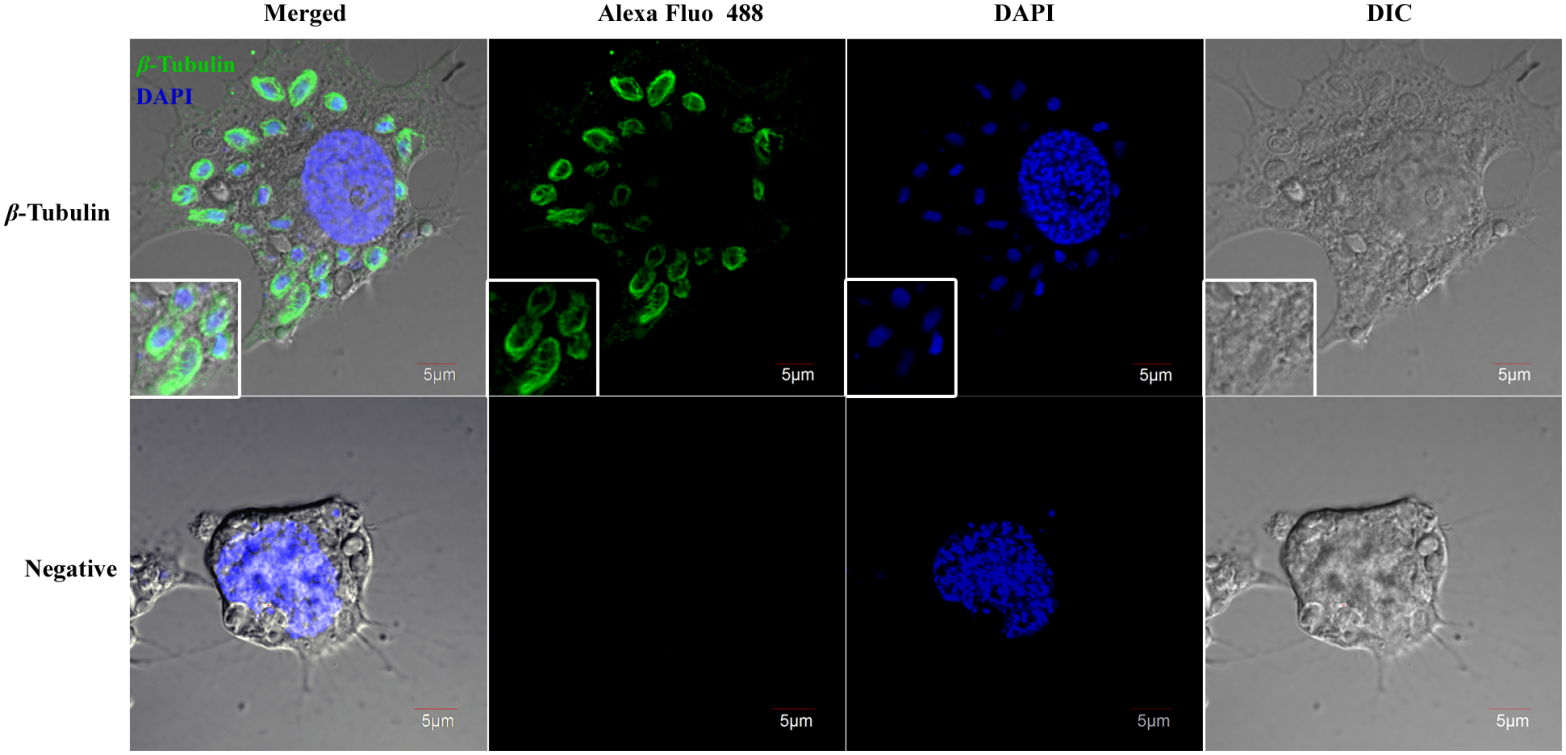
Location of *β*-Tubulin in intracellular microsporidian *N*. *bombycis*. Images were taken by laser scanning confocal microscopy using filter sets for Alexa fluo 488 labeling *β-*Tubulin proteins and DAPI staining nucleus. Immunofluorescence assay with *β-*Tubulin antiserum demonstrated that the membrane and cell plasma location contained in *N*. *bombycis* cells in the proliferative phase. (Bars = 5 μm)

Immunofluorescent assay was used to display the location of *β-*Tubulin at different cell stages. The protein had a non-homogenous distribution throughout the entire parasite cells in the proliferative phase. Tubular fluorescence signals elongated in a cluster to the long axis of meront, while filamentous fluorescence signals formed a mesh architecture. Interestingly, stronger fluorescence was distributed along the cell contour lines near the cell membrane (Fig 2). However, no fluorescence signal was observed in cells in the sporogony phase. This could be due to the gradually thickened spore wall, and antibodies had difficulty in penetrating through the spore wall, and sporoplasm membrane of the parasites in the host cells, which prevented the antibody from finding its target.

### Labeling of microsporidia in the intracellular phase

**Fig 3:**
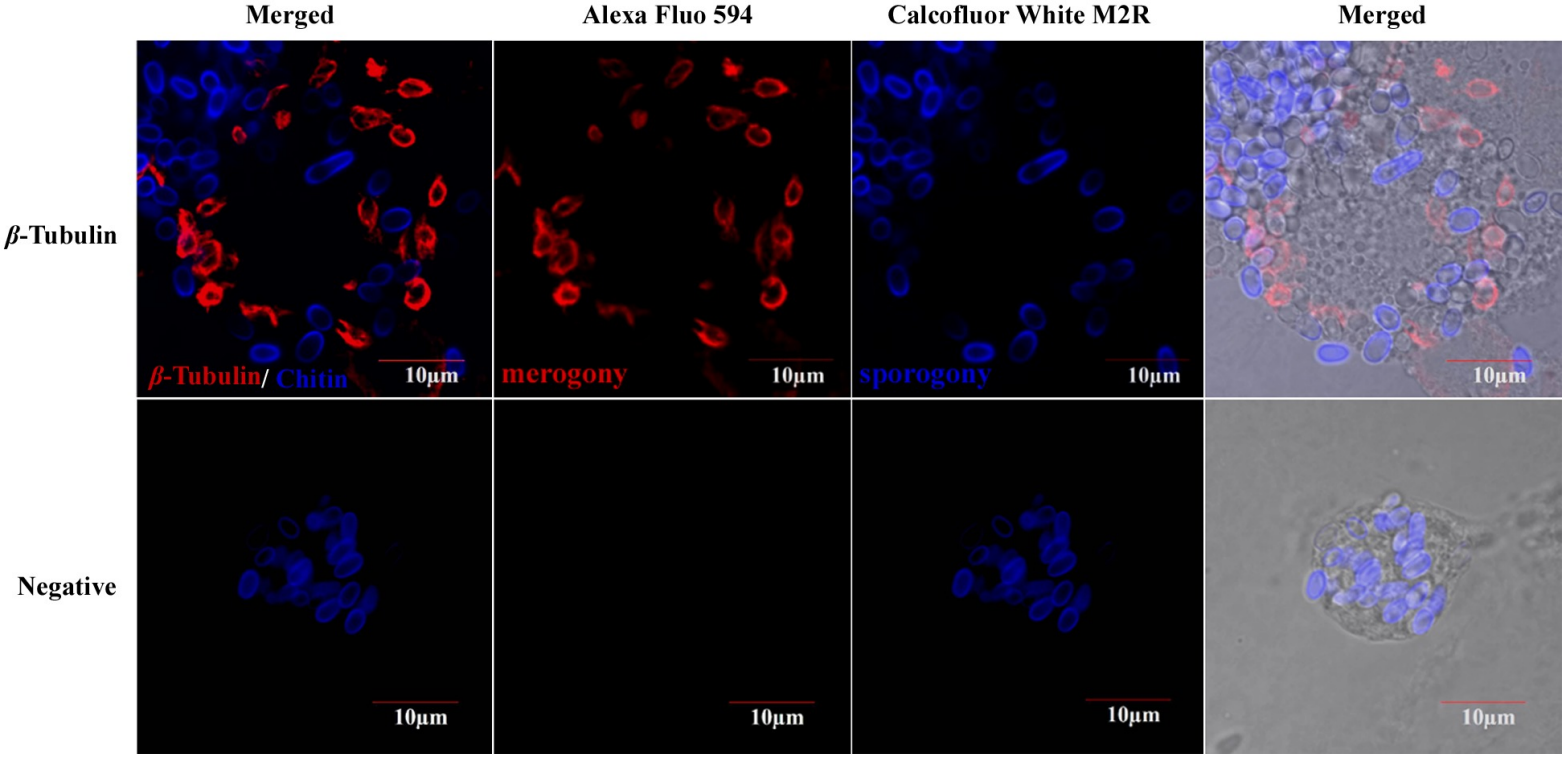
Labeling the merogony phase (red) and the sporogony phase (blue) of microsporidian in *N*. *bombycis*-infected BmE culture cells. Based on the absence or presence of chitin, parasites in proliferative phase were labeled by *β-*Tubulin using an indirect immunofluorescence assay, while cells in sporogonic phase were marked by chitin using 0.1 μg/mL Calcofluor White M2R. *N*. *bombycis* infected culture cells were incubated with negative serum used as a negative control. (Bars = 10 μm).

Based on the different features of microsporidian in different stages in host cells, we constructed a simple labeling method. Red fluorescence that labeled *β-*Tubulin appeared to be more concentrated in the cells of proliferative phase (merogony), while blue fluorescence labeling chitin was only observed on the spore wall in the sporogonic phase (sporogony) (Fig 3). This provides a tool for easily distinguishing the two phases of the microsporidian intracellular cycle.

**Fig 4:**
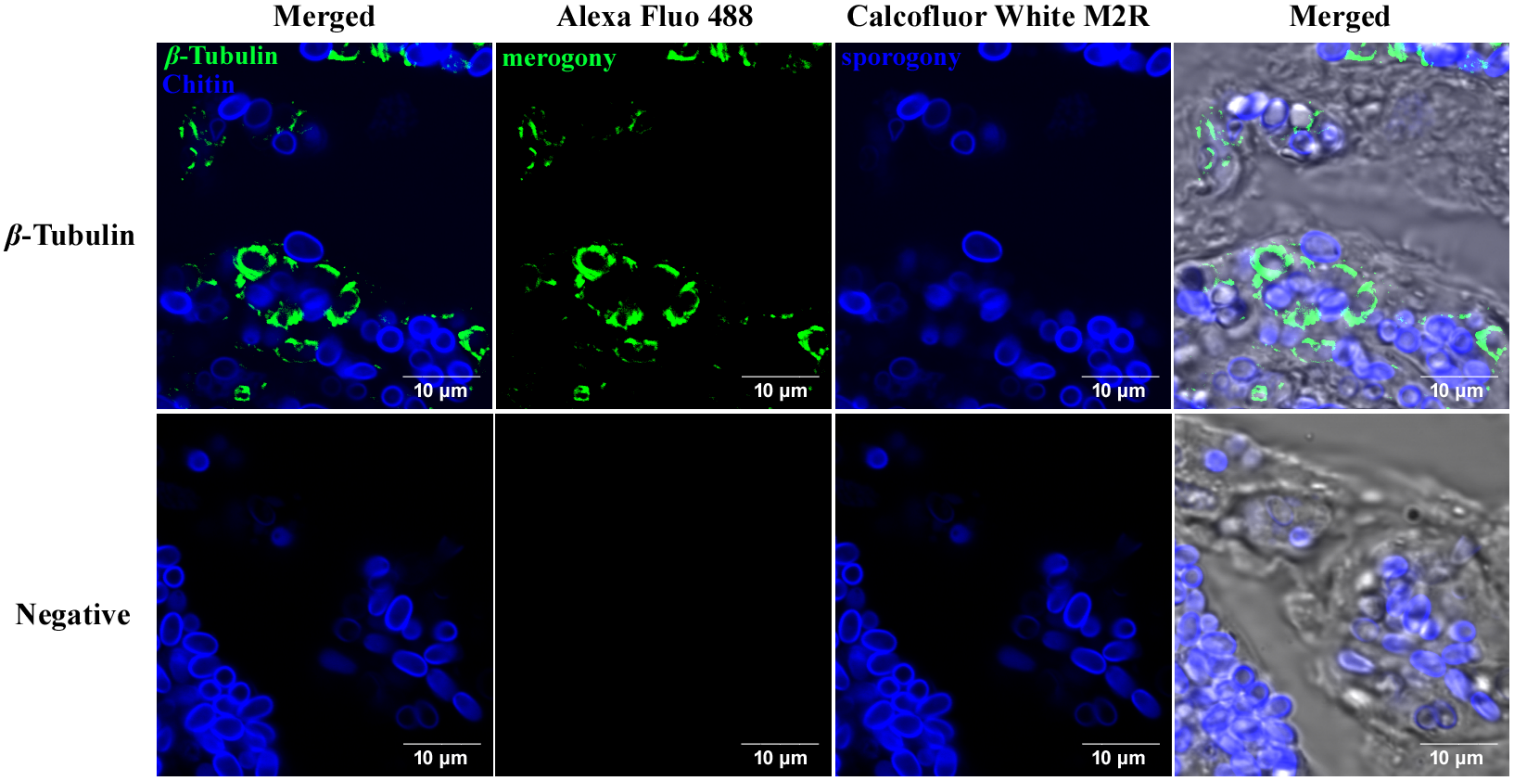
Labeling the two different intracellular phase of microsporidian *N*. *bombycis* by immunofluorescence histochemistry in intestinal tissue slices. *β-*Tubulin antibody coupled with Alexa Fluo 488 (green) labeled secondary antibody were used to label the proliferative phase of microsporidia. Calcofluor White M2R (blue) were used to stain the chitin layer of sporogony phase. (Bars = 10 μm)

Similarly, this method could be used on tissue slices (Fig 4), where, after fixation, permeabilization and antigen retrieval, the histologic section was submitted for immunofluorescence histochemistry. Alternatively, DAPI (S1 Fig) and Calcofluor White M2R can be employed to use together, but if nucleus staining is required, DAPI may be the best dye to use, especially as DAPI fluorescence are usually covered up by chitin-binding dyes.

### Dynamic location of NbSWP12 in the intracellular phase

**Fig 5:**
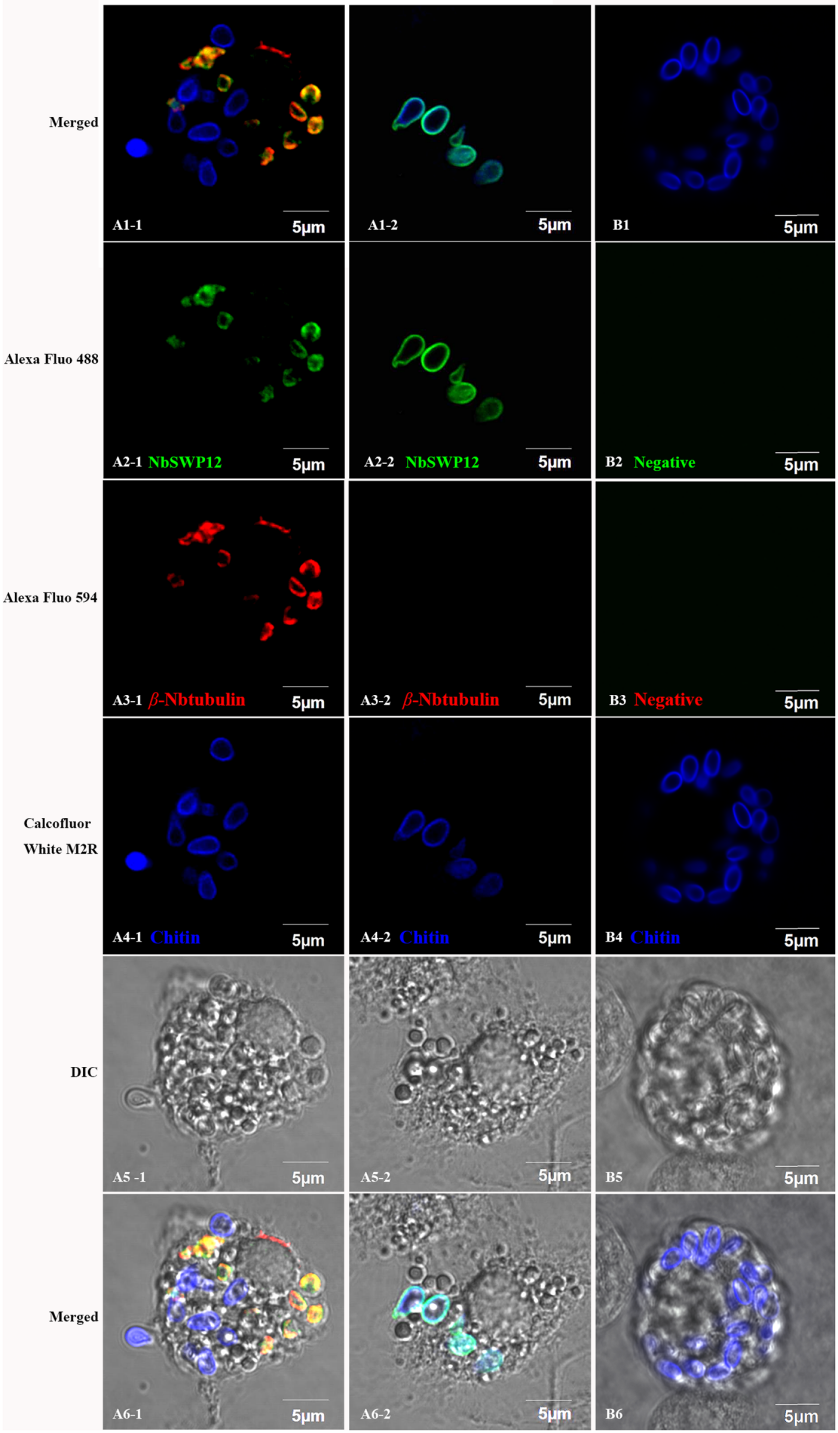
Immunofluorescence localization of NbSWP12 in the intracellular parasite. *N*. *bombycis*-infected BmE cells were incubated with anti-NbSWP12 (A2-1, A2-2) coupled with Alexa Fluo 488 labeled secondary antibody and anti-*β-*Tubulin (A3-1, A3-2) coupled with Alexa Fluo 594 labeled secondary antibody. Red fluorescence of *β-*Tubulin indicated the proliferative phase of *N*. *bombycis*. Blue fluorescence of Calcofluor White M2R-stained chitin displayed the sporogonic phase of *N*. *bombycis*. Overlapping red and green signals (A1-1) indicated that NbSWP12 was partly co-localized with microtubules in the meront. Overlapping green and blue signals (A1-2) in some early sporonts indicated that NbSWP12 was gradually transferred to the spore wall. (Bars = 5 μm).

Previous studies have demonstrated that NbSWP12 is a spore wall protein of *N*. *bombycis* which distribute at exospore, endospore and sporoplasm membrane in mature spores[15, 29]. In order to investigate the protein location of NbSWP12 in the intracellular phase, NbSWP12 polyclonal antibody was used for IFA using this method (Fig 5A1–5A6), while negative mouse and rabbit antiserums were used as the control (Fig 5B1–5B6). The maturity of the parasite’s stages can be assessed by microtubule assembly and chitin presence. The meront is an irregularly-shaped cell that has both filaments and mesh shape characteristics required for microtubule assembly. In the process of chitin layer formation, the parasite eventually transitioned into sporogonic phase. The fluorescence signals of NbSWP12 and *β-*Tubulin indicated NbSWP12 was originally localized at the membrane and partly co-localized with the microtubule in the meront (Fig 5A1-1, 5A2-1 and 5A3-1). Furthermore, Green fluorescence-labeled NbSWP12 could be detected in some early sporonts, which have thin/amorphous chitin layers (Fig 5A1-2, 5A2-2 and 5A3-2).

## Discussion

Microsporidia have been studied for more than 150 years. The structure, evolution, infection and proliferation mechanisms have been interesting questions of scholars for decades. Genome sequencing of microsporidian has led to increased functional research into the organism and its metabolism. However, based on the intracellular parasitization and small size of the organism, most cell cycle and protein locations have been studied using TEM coupled with immunogold labeling or other enhanced methods [11, 21]. In this study we have established a simple yet efficient labeling method that can be performed in most laboratories. Based on the presence of chitin, Calcofluor White M2R or other fluorescent brightener can be used to specifically label the sporogonic phase of microsporidia *N*. *bombycis*. *β-*Tubulin antibody was used to label the proliferative phase of *N*. *bombycis*. Each method has slightly different ranges, which provides enough difference to mark different stages.

Functional research of microsporidia has been a strong research focus for several years, and a series of proteins involved in microsporidian infection or proliferation have been identified [30–33]. ATP/ADP translocases or nucleotide transporters of microsporidia are localized predominantly to the plasma membrane of replicating intracellular cells, where they mediate transport at the host-parasite interface [34, 35]. Yang *et al*. isolated the different life cycle stages of *N*. *bombycis* spores using Percoll gradient centrifugation and displayed SWP9 was secreted to the spore wall prior to SWP7 during the spore development process of *N*. *bombycis*, based on results using IFA and TEM [36]. Subtilisin-like protease 1 of *N*. *bombycis* is mainly localized at the two poles of spore and is only detected in the apical region of the spore coat after germination, which suggests that NbSLP1 plays a significant role in the polar tube extrusion process [26]. AlPTP2b and AlPTP2c of *Antonospora locustae*, NbSWP5, NbSWP11, NbSWP12, NbSWP16 of *N*. *bombycis* and some other proteins of microsporidia have been identified with IFA or TEM in the last five years [15, 37–40]. The localization was significant for protein functional prediction, although the majority of proteins were observed in mature spore or the sporogonic phase via IFA or TEM. Although some protein localizations in the intracellular parasites were identified by the immunofluorescent assay, specific stages remain uncertain. In combination with another antibody, this method can readily demonstrate the location of the protein in cells from different stages. At present, the method has been applied to study a variety of protein localizations. The dynamic position of proteins can be easily observed by this method. Due to the relatively impermeable spore wall of the pathogen while it infests host cells, antibodies were difficult to combine with the antigen in the sporoplasm during the sporogonic phase. That is why the *β-*Tubulin can be used to mark the proliferative phase of *N*. *bombycis*. We then used this method to investigate the intracellular location of NbSWP12. The spore wall protein is a predicted Bin/Amphipysin/Rvs (BAR) domain protein which distribute at exospore, endospore and sporoplasm membrane of mature spores [15, 29, 41]. No intracellular location information of this protein was reported. The experiment result indicated NbSWP12 distribute near the membrane and partly co-localized with microtubule. Considering NbSWP12 maybe a member of BAR superfamily that mostly act as membrane-molding macromolecules, it is likely that the protein and microtubule proteins may have synergistic effects on cell membrane. NbSWP12 then gradually labeled the spore wall with a fluorescent ring during the maturation process of *N*.*bombycis*. Previous study showed the protein could adhere with the deproteinized chitin coat of the parasite [15]. Combined with the location in the sporogny phase and mature spores, the protein may function in spore wall maintenance at endospore and might shape the host cell membrane curvature to help the parasite to be phagocytosis at exospore. We have evaluated the methods and proved the efficiency and feasibility.

Santiana *et al*., reported a fluorescent labeling method that enables monitoring the dynamic of developing microsporidia based on the vital staining of nuclei and conjunctions with fluorescent protein-tagged host proteins [42]. Dubuffet *et al*., designed fluorescenct *in situ* hybridization probes for two distinct microsporidian clades and demonstrated their efficacy in detecting and studying the vertical transmission of two microsporidia [22]. These methods are ideal for microsporidian visualization but not applicable for display protein location during the development of the parasite. Our method described in this paper also provides good methodologies in cell cycle research and detecting parasites. In addition, the *β-*Tubulin antibody can be replaced by some other antibodies that the location of the antigen have been clearly known to help uncover subcellular localization of a novel protein. Furthermore, it can be applied in dynamic morphological study of the intracellular phase. *N*. *bombycis* possess diplokaryon in which two nuclei coexist within a cell and are appressed to one another [43]. But how the diplokaryon formed during the development is still unclearly. Using the method that we report, an antibody recognizing the karyolemma of *N*. *bombycis* can be prepared and combine with the nuclei dye, the dynamic processes of nuclear division and cell proliferation in host cells can be observed.

## Conclusions

Using chitin stain dye Calcofluor White M2R and *β-*tubulin antibody coupled with fluorescence secondary antibody, this study represents an important method in differentiating proliferative and sporogonic phases of Microsporidia *N*. *bombycis* and provides an easy technique to visualize parasite infection and protein location in the intracellular phase. This process is simple and economical and has great application value in microsporidia research.

## Supporting information

S1 Fig Labeling the proliferative phase of microsporidian by immunofluorescence histochemistry in oviduct epithelial tissue slices. *β-*Tubulin antibody coupled with Alexa Fluo 594 labeled secondary antibody was used to label the proliferative phase of microsporidia. DAPI (blue) were used to stain the nucleus. (Bars = 10 μm).
(TIF)

S1 Text Sequence of *N*.*bombycis β-*tubulin. (TXT)
