# Supplementary figures and images for: Easy labeling of proliferative phase and sporogonic phase of microsporidia Nosema bombycis in host cells
Source: PLoS One. 2017 Jun 22;12(6):e0179618. doi: 10.1371/journal.pone.0179618 (PMC5480951; doi:10.1371/journal.pone.0179618)

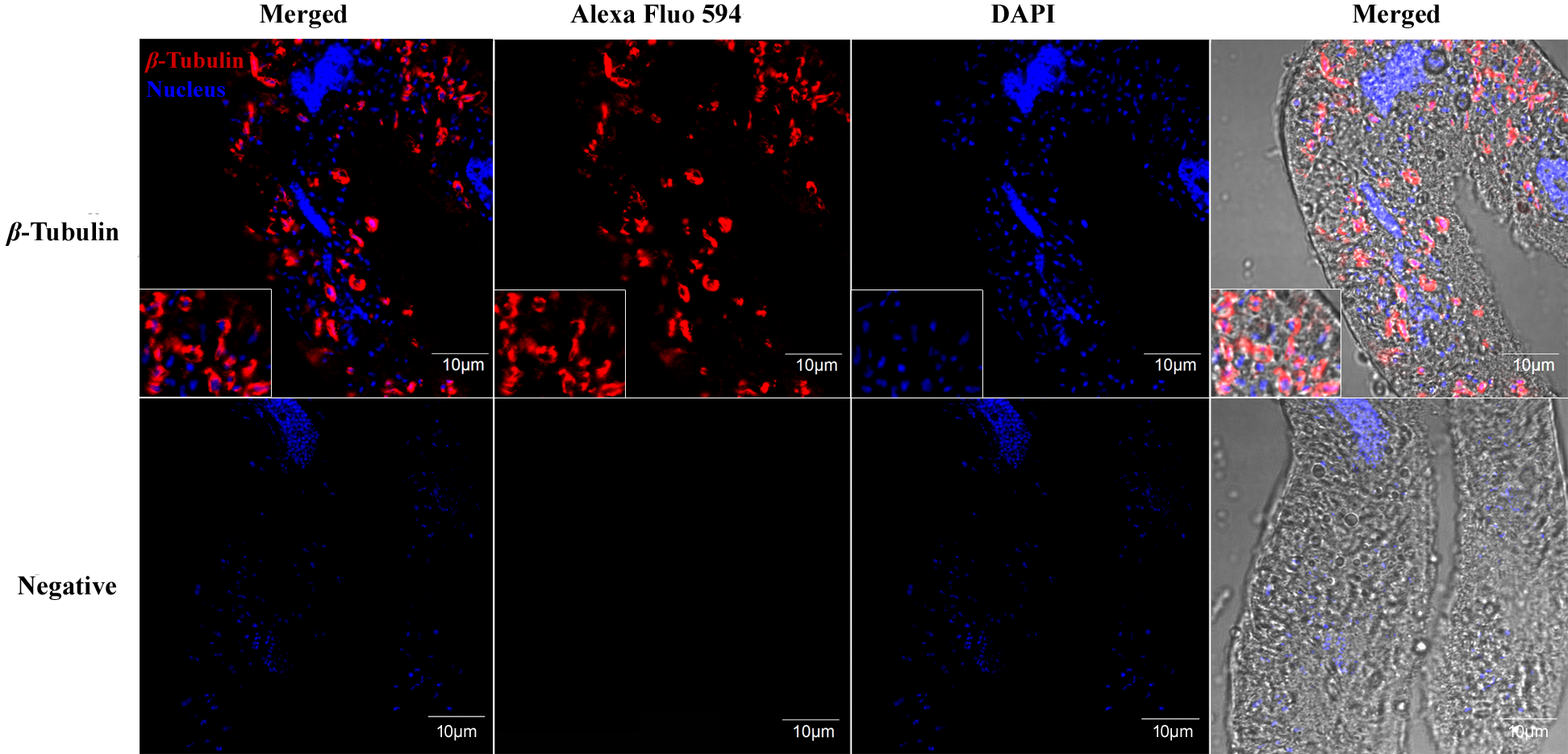

Supplement: S1 Fig — β-Tubulin antibody coupled with Alexa Fluo 594 labeled secondary antibody was used to label the proliferative phase of microsporidia. DAPI (blue) were used to stain the nucleus. (Bars = 10 μm). (TIF) [file pone.0179618.s001.tif]
